# Supplementary material for: Protein aggregation is a consequence of the dormancy-inducing membrane toxin TisB in Escherichia coli
Source: mSystems. 2024 Oct 8;9(11):e01060-24. doi: 10.1128/msystems.01060-24 (PMC11575346; doi:10.1128/msystems.01060-24)
Supplement: Supplemental Figures — Figures S1 to S9. [file msystems.01060-24-s0003.pdf]

Supplemental Figures

Protein aggregation is a consequence of the dormancy-inducing membrane toxin TisB in *Escherichia coli*

Florian H. Leinberger, Liam Cassidy, Daniel Edelmann, Nicole E. Schmid, Markus Oberpaul, Patrick Blumenkamp, Sebastian Schmidt, Ana Natriashvili, Maximilian H. Ulbrich, Andreas Tholey, Hans-Georg Koch and Bork A. Berghoff

This file contains:

Figures S1 to S9

Figure S1

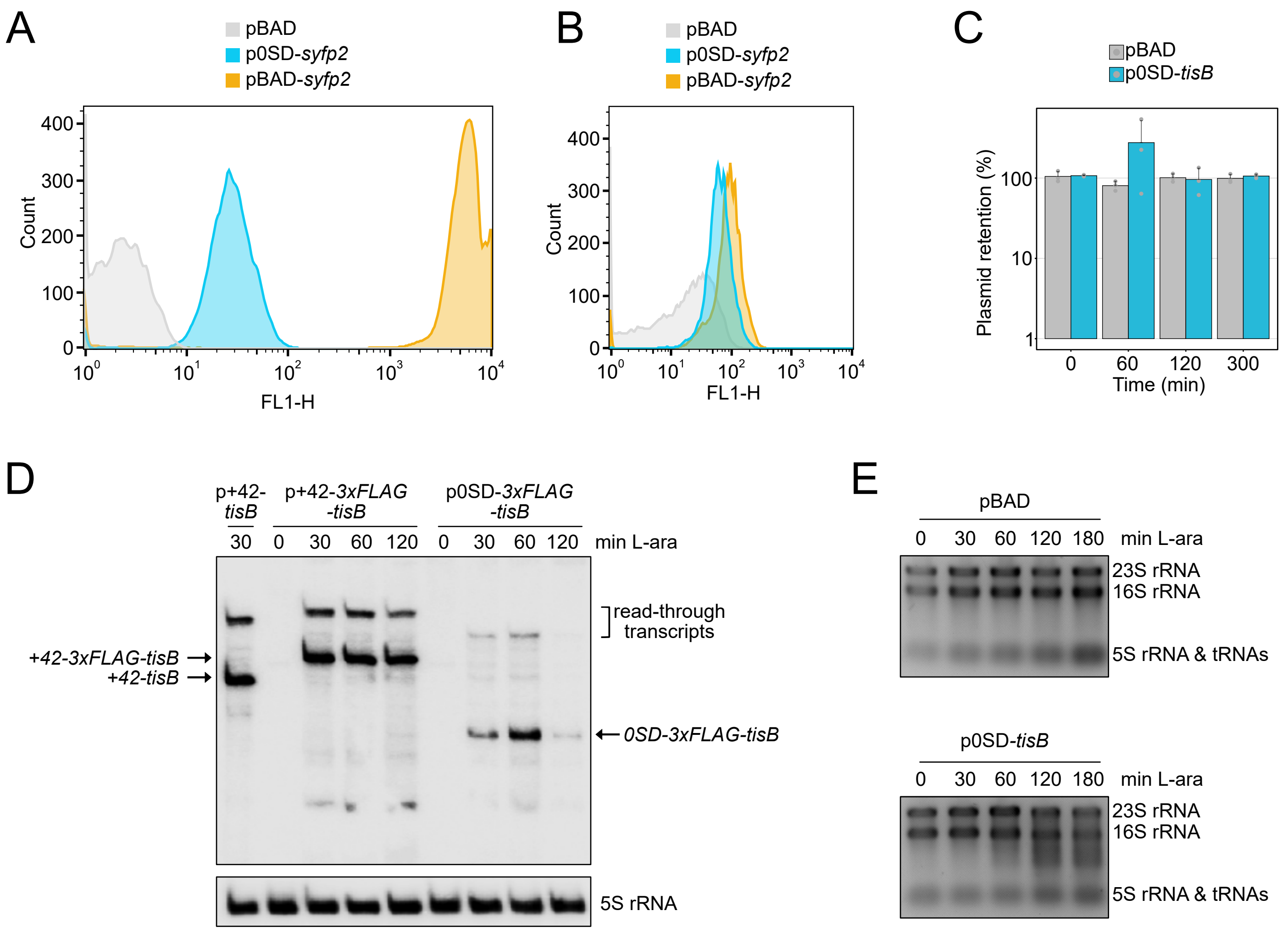

Figure S1. Evaluation of the p0SD-*tisB* expression system.

(A) Translational efficiency of the SD-free 5' UTR (0SD). Wild type MG1655, harboring either an empty pBAD plasmid, p0SD-*syfp2* or pBAD-*syfp2*, was treated with L-ara (0.2%) during the exponential phase (OD_{600} ~ 0.4) for 1 hour. Samples were resuspended in 100 µl paraformaldehyde (2% in 1x PBS) and incubated in the dark at room temperature for 10 minutes. Samples were washed two times with 200 µl 1x PBS before resuspended in 250 µl 1x PBS. The sYFP2 fluorescence was measured using flow cytometry and detecteor FL1-H. **(B)** Same samples as in (A). sYFP2 fluorescence was measured using flow cytometry at various detector intensities to ensure that the data is centered: empty pBAD plasmid (grey | 999), p0SD-*syfp2* (blue | 774) and pBAD-*syfp2* (orange | 412). **(C)** Plasmid stability. Wild type MG1655, harboring either p0SD-*tisB* or an empty pBAD plasmid, was treated with L-ara (0.2%) during the exponential phase (OD_{600} ~ 0.4). Samples were collected at specified time points and plated on LB agar plates, both with and without 200 µg/ml ampicillin. Cell counts were used to calculate the relative plasmid retention. Bars represent the mean of three biological replicates and error bars indicate the standard deviation. Dots show individual data points. **(D)** Northern blot analysis of different *tisB* expression systems. Wild type MG1655, harboring pBAD derivatives with different *tisB* fragments with and without 3xFLAG sequence, was grown to an OD_{600} of ~0.4 (exponential phase) and treated with L-arabinose (L-ara; 0.2 %) as inducer. Samples were collected at the indicated time points for total RNA isolation and subsequent northern blot analysis. A radioactively labelled probe, targeted against the *tisB* open reading frame, was used for detection of *tisB* transcripts. 5S rRNA was probed as loading control. **(E)** Integrity of abundant RNAs. Wild type MG1655, harboring an empty pBAD plasmid or p0SD-*tisB*, were grown to an OD_{600} of ~0.4 (exponential phase) and treated with L-ara (0.2 %). Samples were collected at the indicated time points for total RNA isolation and subsequent quality analysis on 1% agarose gels, containing 1x TBE and 25 mM guanidinium thiocyanate.

Figure S2

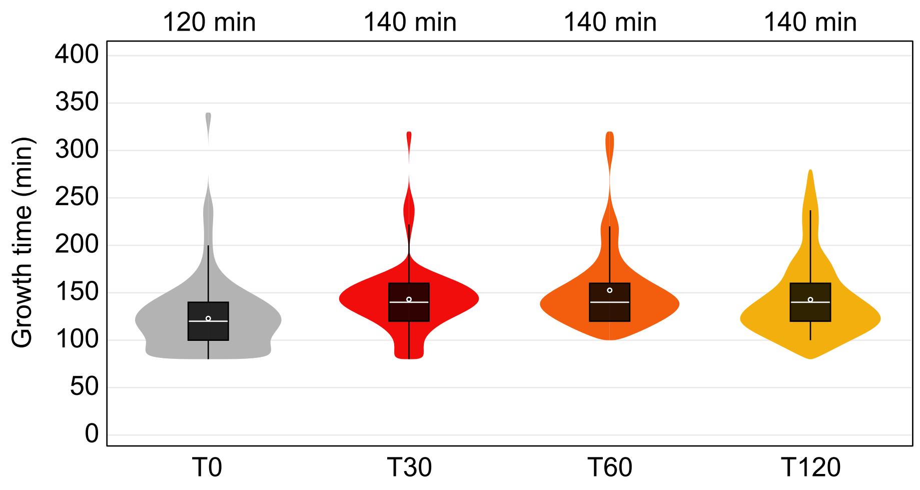

Figure S2. Colony growth time analysis by ScanLag.

ScanLag analysis was applied to determine the colony growth time after *tisB* expression. For each time point, colony growth times are illustrated as violin box plots. Colonies from three biological replicates were combined (T0: n=154; T30: n=59; T60: n=103; T120: n=124). The white dot indicates the mean. The corresponding median appearance time (white bar) is shown on top of each plot.

Figure S3

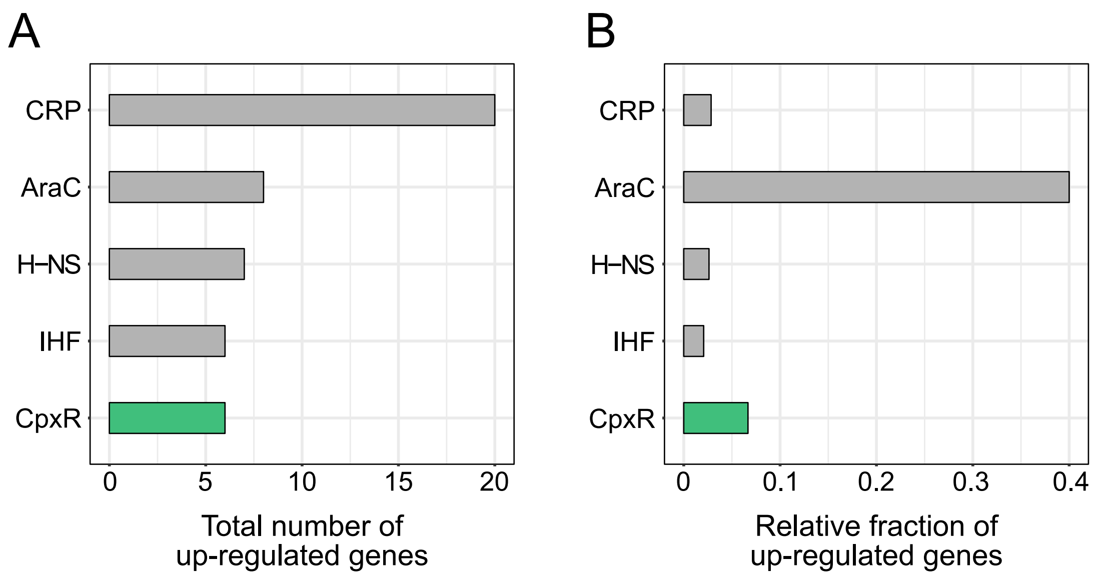

Figure S3. Regulon analysis of TisB-responsive genes.

RNA-seq identified 67 genes that were up-regulated upon *tisB* expression using the p0SD-*tisB* system (log_2 fold change >2 and p-value <0.01). These genes were subjected to a regulon analysis using Bioconductor package regutools (R package for data extraction from RegulonDB). **(A)** Top-5 regulons according to total numer of up-regulated genes. **(B)** Top-5 regulons according to the numer of up-regulated genes in relation to the regulon size.

Figure S4

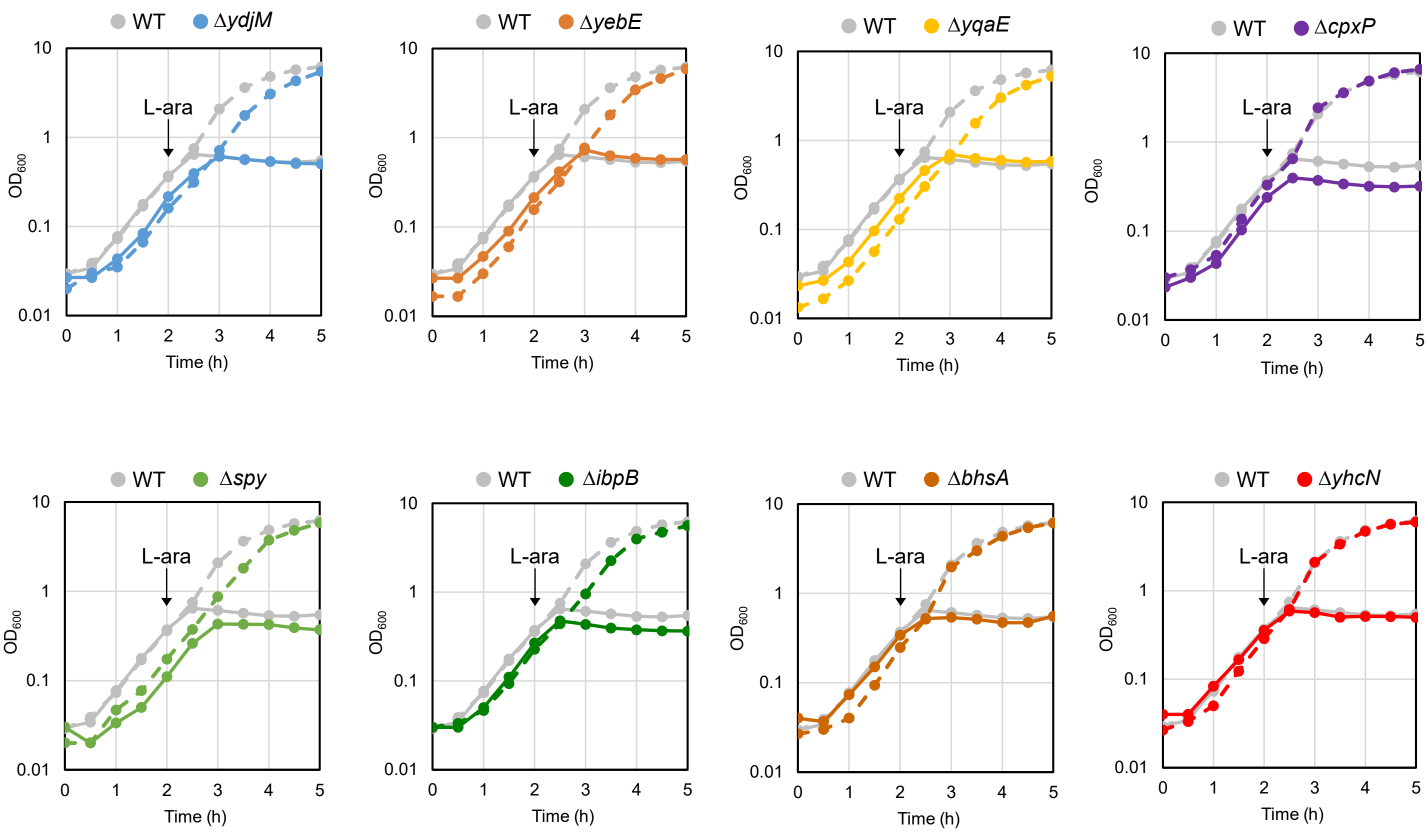

Figure S4. TisB-induced growth inhibition in different deletion mutants.

MG1655 wild type (WT) and isogenic deletion mutants, containing either an empty pBAD plasmid (dashed curves) or p0SD-*tisB* (solid curves), were grown in LB medium at 37°C. The OD_{600} was monitored over time. At the indicated time point during exponential phase, cultures were treated with L-arabinose (L-ara; 0.2%) as inducer.

Figure S5

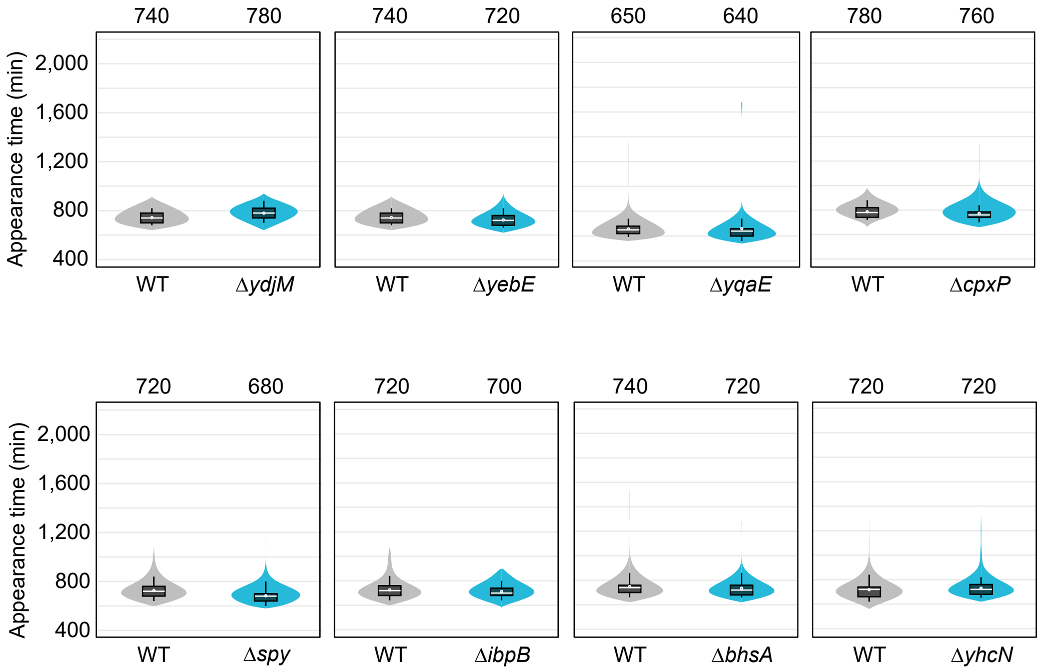

Figure S5. ScanLag analysis of selected deletion mutants.

Wild type (WT) MG1655 and deletion mutants, harboring the empty pBAD plasmid, were treated with L-ara (0.2%) during exponential phase (OD_{600} ~0.25) for 1 hour. ScanLag was applied to determine the colony appearance time after L-ara treatment. For each deletion mutant, colony appearance times are illustrated as violin box plots and compared to a corresponding wild type. Colonies from at least three biological replicates were combined (WT: n≥91; Δ*ydjM*: n=332; Δ*yebE*: n=265; Δ*yqaE*: n=75; Δ*cpxP*: n=84; Δ*spy*: n=263; Δ*ibpB*: n=197; Δ*bhsA*: n=180; Δ*yhcN*: n=293). The white dot indicates the mean. The respective median appearance time (white bar) is shown on top of each plot.

Figure S6

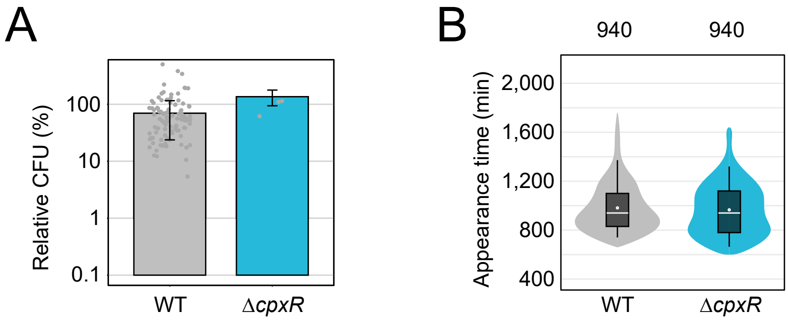

Figure S6. Analysis of the *cpxR* deletion mutant.

Wild type (WT) MG1655 and the Δ*cpxR* mutant, harboring plasmid p0SD-*tisB*, were treated with L-ara (0.2%) during exponential phase (OD_{600} ~0.4) for 1 hour. **(A)** Pre- and post-treatment samples were used to determine relative CFU (%). Bars represent the mean of at least three biological replicates and error bars indicate the standard deviation. Dots show individual data points. **(B)** ScanLag was applied to determine the colony appearance time after L-ara treatment. Colony appearance times are illustrated as violin box plots (WT: n=235; Δ*cpxR*: n=185). The white dot indicates the mean. The respective median appearance time (white bar) is shown on top of each plot.

Figure S7

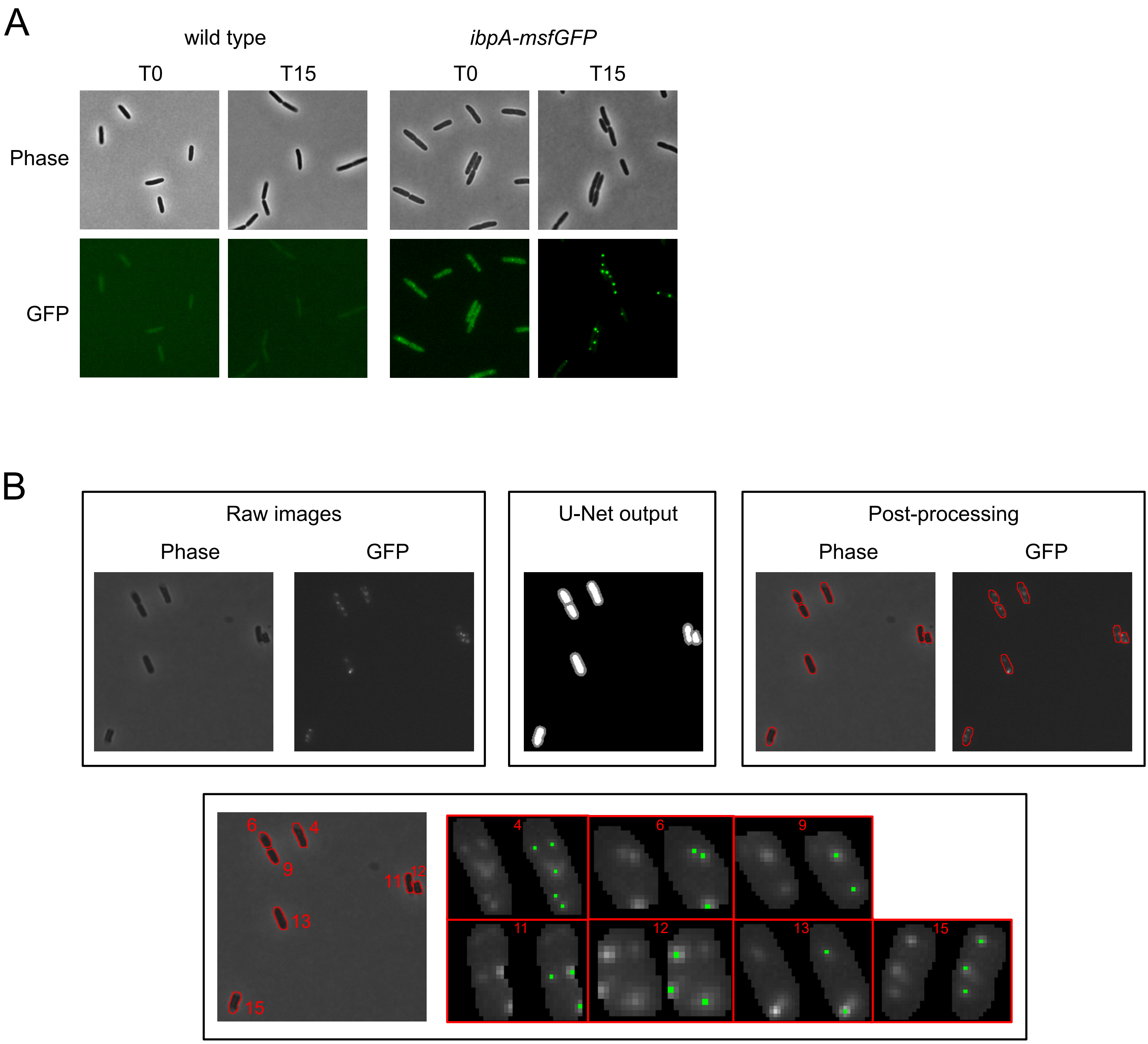

Figure S7. Validation of the *ibpA-msfGFP* reporter and U-Net analysis of msGFP foci.

(A) Wild type MG1655 and reporter strain *ibpA-msfGFP* were grown to exponential phase (T0; OD_{600} ~0.4) and shifted to 47°C for 15 minutes (T15). Phase contrast images are displayed together with corresponding fluorescence images (GFP). **(B)** Exemplary representation of msfGFP foci counting using U-Net. Upper panel: Raw images from phase contrast and fluorescence images (GFP) are applied to post-processing using the U-Net model output for cell segmentation. Lower panel: According to cell shapes, GFP images of single cells are extracted and msfGFP foci are determined.

Figure S8

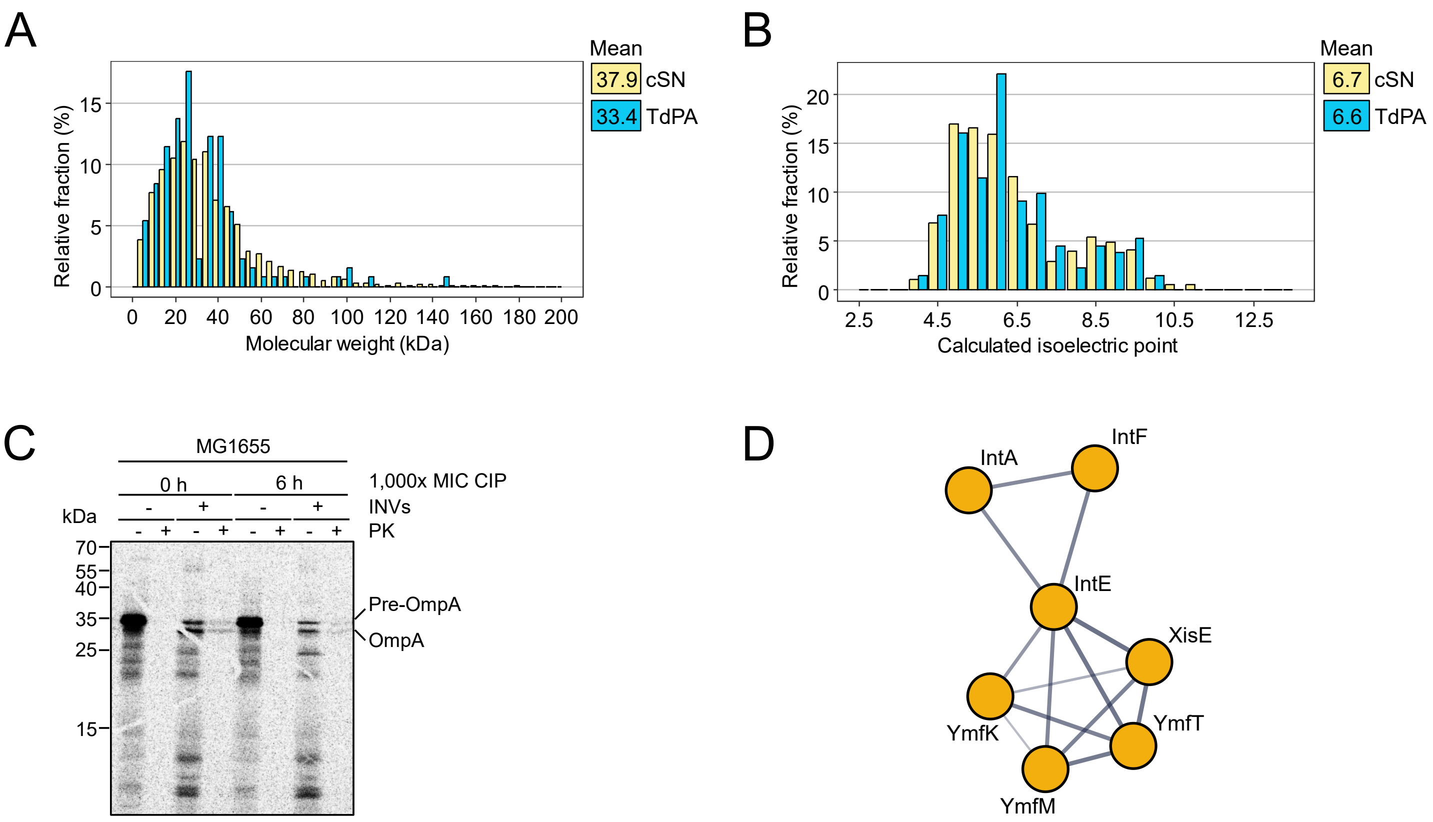

Figure S8. Analysis of protein aggregates and inner membrane vesicles.

(A) Distribution plot of the molecular weight (kDa). The relative fractions are shown for the combined supernatant (cSN) and TisB-dependent protein aggregates (TdPA). Means are displayed in the legend. **(B)** Distribution plot of the calculated isoelectric point. The relative fractions are shown for the combined supernatant (cSN) and TisB-dependent protein aggregates (TdPA).Means are displayed in the legend. **(C)** Cell-free OmpA synthesis was performed in a coupled transcription/translation system using T7 RNA polymerase and CTF extract with purified ribosomes. Reactions were prepared on ice and translation was carried out in the presence of radioactive ^{35}S-methionine/^{35}S-cysteine labelling mix (Perkin Elmer Wiesbaden, Germany). Radioactively labelled OmpA was used in in-vitro translocation reaction with two different inner membrane vesicles (INVs). In one case *E. coli* K-12 wild type MG1655 cells were harvested for membrane preparation at an OD_{600} of 1.2-1.6 (0 h), while in the other case *E. coli* K-12 wild type MG1655 cells were treated with 1,000x MIC of ciprofloxacin (CIP) for six hours (6 h), starting at an OD_{600} of 0,4. In order to asses the translocation of OmpA across the inner membrane, after synthesis it was incubated with INVs (0,1 µg protein) for 25 minutes at 37°C and treated with Proteinase K (PK) (0.2 mg/ml PK for 30 minutes at 25℃). INVs were not added to the control reactions in order to show complete digestion of OmpA with PK in the absence of INVs. All samples were precipitated with 5% TCA for at least 30 minutes, separated on SDS-PAGE and analyzed by autoradiography. Indicated are pre-OmpA and mature OmpA, which occurs after signal sequence cleavage. (D) Protein-protein association network of prophage proteins in TisB-dependent protein aggregates (TdPA). The network was revealed by a multi-protein search using the STRING database (https://string-db.org/), indicating the enrichment of the corresponding proteins within the category ‘viral process and bacteriophage tail fiber assembly’. The thickness of connecting lines indicates the confidence of the respective interaction.

Figure S9

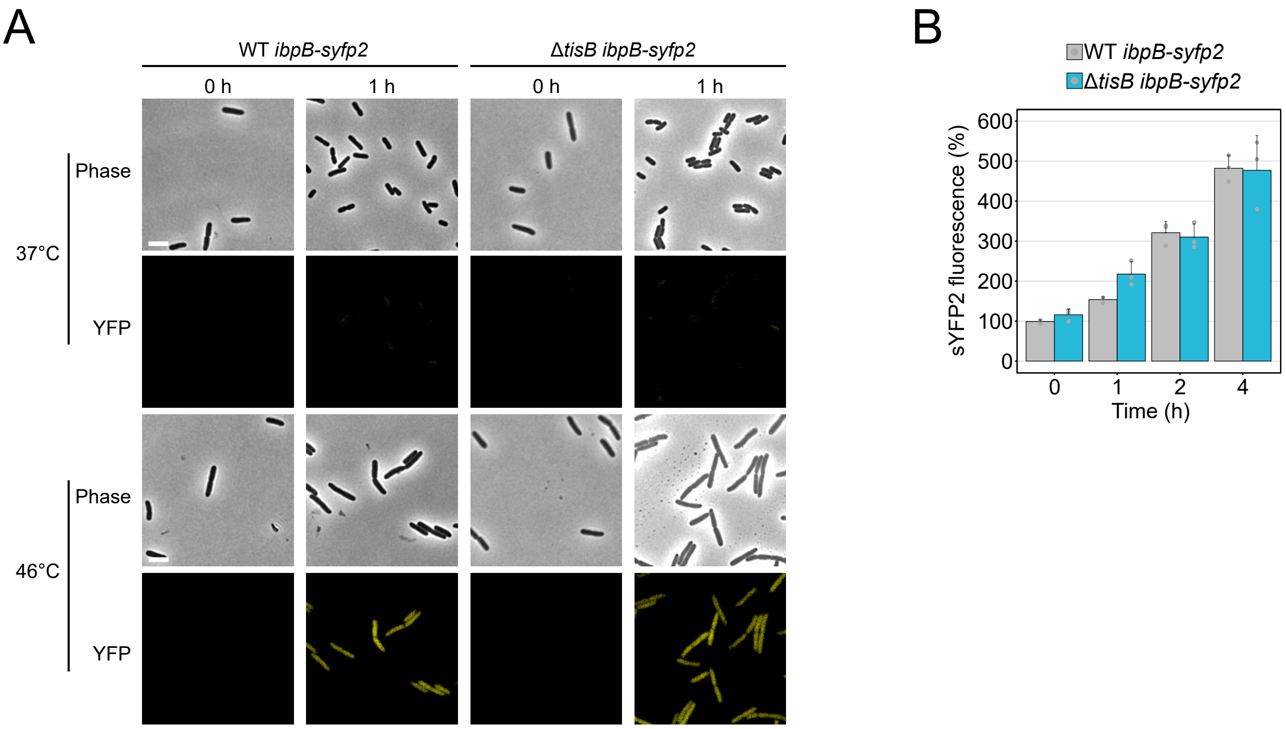

Figure S9. Induction of the heat shock response.

(A) Wild type (WT) *ibpB-syfp2* and Δ*tisB ibpB-syfp2* were grown to an OD_{600} of ~0.4 at 30°C and subsequently shifted to 37°C and 46°C. Samples were collected before (0 h) and after (1 h) the temperature shift and analyzed by microscopy. Phase contrast images are displayed together with corresponding fluorescence image (YFP). White bars represent a length scale of 2 µm. **(B)** Wild type (WT) *ibpB-syfp2* and Δ*tisB ibpB-syfp2* were grown to an OD_{600} of ~0.4 at 30°C and subsequently shifted to 37°C and 46°C. Samples were collected before (0 h) and after (1 h, 2 h, 4 h) the temperature shift. Fluorescence was measured using a plate reader, and the relative sYFP2 fluorescence was calculated in comparison to the fluorescence at 37°C. Bars represent the mean of three biological replicates and error bars indicate the standard deviation. Dots show individual data points.
